# Intensive Mutagenesis of the Nisin Hinge Leads to the Rational Design of Enhanced Derivatives

**DOI:** 10.1371/journal.pone.0079563

**Published:** 2013-11-11

**Authors:** Brian Healy, Des Field, Paula M. O’Connor, Colin Hill, Paul D. Cotter, R. Paul Ross

**Affiliations:** 1 School of Microbiology, University College Cork, Cork, Ireland; 2 Alimentary Pharmabiotic Centre, University College Cork, Cork, Ireland; 3 Teagasc Food Research Centre, Moorepark, Fermoy, County Cork, Ireland; Russian Academy of Sciences, Institute for Biological Instrumentation, Russian Federation

## Abstract

Nisin A is the most extensively studied lantibiotic and has been used as a preservative by the food industry since 1953. This 34 amino acid peptide contains three dehydrated amino acids and five thioether rings. These rings, resulting from one lanthionine and four methyllanthionine bridges, confer the peptide with its unique structure. Nisin A has two mechanisms of action, with the N-terminal domain of the peptide inhibiting cell wall synthesis through lipid II binding and the C-terminal domain responsible for pore-formation. The focus of this study is the three amino acid ‘hinge’ region (N 20, M 21 and K 22) which separates these two domains and allows for conformational flexibility. As all lantibiotics are gene encoded, novel variants can be generated through manipulation of the corresponding gene. A number of derivatives in which the hinge region was altered have previously been shown to possess enhanced antimicrobial activity. Here we take this approach further by employing simultaneous, indiscriminate site-saturation mutagenesis of all three hinge residues to create a novel bank of nisin derivative producers. Screening of this bank revealed that producers of peptides with hinge regions consisting of AAK, NAI and SLS displayed enhanced bioactivity against a variety of targets. These and other results suggested a preference for small, chiral amino acids within the hinge region, leading to the design and creation of producers of peptides with hinges consisting of AAA and SAA. These producers, and the corresponding peptides, exhibited enhanced bioactivity against *Lactococcus lactis* HP, *Streptococcus agalactiae* ATCC 13813, *Mycobacterium smegmatis* MC2155 and *Staphylococcus aureus* RF122 and thus represent the first example of nisin derivatives that possess enhanced activity as a consequence of rational design.

## Introduction

Bacteriocins are small, bacterially produced, ribosomally synthesized peptides that are active against other bacteria and against which the producer has a specific immunity mechanism [1], [2]. The bacteriocins can be subdivided on the basis of their structure with Class 1 consisting of peptides that have undergone post-translational modification [3]. Of these, the lantibiotics have been the focus of particular attention [4], [5]. The name of these bacteriocins reflects their structure; ‘**Lan** thionine-containing an**tibiotics**’, where lanthionines/β-methyllanthionines are unusual residues that are formed between cysteines and neighbouring dehydrated serines (dehydroalanines) or threonines (dehydrobutyrines), respectively [5].

**Figure 1:**
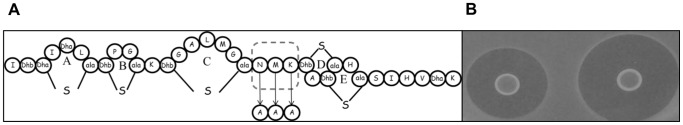
A - Nisin A mature peptide, B - AAA Producer (Deferred Antagonism Assay). The structure of the mature nisin A peptide and the zone of inhibition exhibited by the nisin A derivative, AAA. Panel A depicts the amino acid changes that produce the AAA derivative in the nisin ‘hinge’ region. The modified amino acids dehydroalanine and dehydrobutyrine are denoted as Dha and Dhb respectively with the five (β-methyl)lanthionine rings labelled A to E. Panel B depicts the zones of inhibition produced by *L. lactis* NZ9800 pDF05(left) and AAA ‘hinge’ variant (right) against *L. lactis* HP.

The lantibiotic Nisin A (Fig. 1) has been used a commercial food additive since 1953 and has been approved for use in food by the FAO, WHO, EU and the USFDA [1], [6]. Unsurprisingly, Nisin A is by far the most extensively studied lantibiotic [1], [6], [7]. In addition, the gene encoded nature of nisin, and indeed other lantibiotics, can be exploited through engineering to even further enhance its activity and/or investigate structure-function relationships. Such engineering can be carried out *in vivo* (manipulation of the producer or heterologous expression of the genes in another host) or *in vitro* (using purified components of the biosynthetic machinery) [8]–[11].

The *in vivo* engineering of nisin through the replacement of specific amino acids commenced in 1992 [12] and a number of derivatives were identified that have been of considerable value with respect to revealing the fundamentals of nisin biology [13], [14]. More recently, derivatives have been identified with academic and potential commercial value that display enhanced activity against pathogenic bacteria [8], [15]–[19]. The majority of these enhanced derivatives differ with respect to the amino acids found in the 3 amino acid ‘hinge’ region of the peptide. This region is thought to be key with respect to linking the two functional domains of the nisin peptide and providing conformational flexibility between these regions [20], [21]. Nisin inhibits cell wall synthesis through the formation of a complex with lipid II, an essential precursor of peptidoglycan synthesis [22], [23], with the N-terminus region being responsible for this binding [24]. The hinge links this domain with the C-terminal end of the peptide, which is responsible for a second mechanism of antimicrobial activity, which involves permeabilization of the cell membrane. The first indication that mutagenesis of the hinge could bring about beneficial consequences was provided by Yuan and co-workers [18] who established that two derivatives of nisin Z (N20K and M21K), exhibited enhanced activity against Gram negative (*Shigella, Peudomonas* and *Salmonella*), but not Gram positive, pathogens. This was followed by the identification of hinge derivatives, such as N20P, M21V, K22S and K22T [8], with enhanced activity against Gram positive pathogens (*Streptococcus agalactiae*, *Staphylococcus aureus* and *Listeria monocytogenes*). Further studies have emphasised the enhanced potency of one of these variants, nisin V (M21V) [25], against a broad variety of drug-resistant pathogens and have identified other hinge derivatives, such as that containing the residues SVA, that exhibit enhanced activity in complex matrices [17].

The hinge derivatives which have been studied to date have resulted from strategies in which one or two of the hinge residues have been manipulated. Here we go a step further through the randomisation of all three hinge residues simultaneously. Screening of a bank of producers of such derivatives revealed a pattern whereby many producers of derivatives containing small chiral amino acids within the hinge displayed enhanced bioactivity. This prompted the rational design of additional derivatives not identified from the random bank, peptides containing AAA and SAA hinges, which were particularly notable with respect to the extent to which bioactivity was enhanced relative to the wild type producer.

## Experimental Procedures

### Bacterial strains and growth conditions

All strains and plasmids used in this study are listed in Table 1. *Lactococcus lactis* cultures were grown in M17 broth or agar (1.5%) (Oxoid) supplemented with 0.5% glucose at 30°C. *Escherichia coli* strains were grown in Luria-Bertani (LB) broth or agar with continuous shaking at 37°C. Where necessary, chloramphenicol was used at 10 μg/ml for *L. lactis* and *E. coli*. *Staphylococcus aureus* RF122 was grown in Brain Heart Infusion (BHI) broth or agar (Oxoid) at 37°C. *Streptococcus agalactiae* ATCC 13813 was grown in Tryptic Soy Broth (TSB) (Merck) or TS agar supplemented with Yeast Extract (YE) (Oxoid) at a concentration of 0.6% at 37°C. *Mycobacterium smegmatis* MC2155 was grown on Middlebrook 7H9 broth (BD) or Middlebrook 7H10 agar (BD) supplemented with 0.05% tween 80 and 2% glycerol at 37°C.

**Table 1:** Plasmids & strains used in used in this study.

Plasmid/Strains	Characteristic	Reference/Source
pDF05	pCI372 with *nis*A	[8]
pDF05 AAK	pDF05 with N20A/M21A substitution in *nis*A	This study
pDF05 AAA	pDF05 AAK with K22A substitution in *nis*A AAK	This study
*L. lactis* NZ9800	*L. lactis* NZ9700 Δ*nisA*	[34]
*L. lactis* NZ9800 pDF05	Wild type nisin A producer	[8]
*E. coli* Top10	Intermediate cloning host	Invitrogen
*M. smegmatis* MC2155	Model microorganism for slow-growing mycobacteria species	ATCC
*S. agalactiae* ATCC 13813	Indicator strain	ATCC
*L. lactis* ssp. *cremoris* HP	Indicator strain	UCC Culture Collection
*S. aureus* RF122	Bovine mastitis-causing isolate	[35]

### Site-saturation mutagenesis of the nisin A ‘hinge’ region

The complete randomisation of the ‘hinge’ amino acids was achieved by PCR using the template pDF05 (pCI372–*nisA*) and primers NisAXXXHingeFor and NisAXXXHingeRev (Table 2). The template DNA was extracted using a High Pure Plasmid Isolation Kit (Roche) from dam^+^ *E. coli* Top10 (Invitrogen) to ensure its methylation. PCR amplification was performed in 50 µl volumes with 1 ng per 50 µl of template, 2 units of Phusion High Fidelity DNA Polymerase (Finnzymes), approximately 0.5ng of template, 1x HF buffer, 200 µM dNTPs and 0.5 µM of the relevant oligonucleotides. Cycling conditions were as follows: 98°C for 30 secs, 55°C for 15 secs, 72°C for 3.5 mins for 40 cycles followed by final extension for 10 mins. Samples were PCR cleaned using GeneJet PCR Purification Kit (Thermo Scientific) followed by *Dpn*I treatment (Stratagene) at 37°C for 3 hours. 2.5 µl of mutated pCI372-NisA was added to a vial of Top 10 Chemically Competent *E. coli* (Invitrogen). These transformants were pooled, plasmids extracted using a High Pure Plasmid Isolation Kit (Roche) and transformed into electro-competent. *L. lactis* ssp. *cremoris* NZ9800▵*nisA* and selected for on GM17 Cm^10^ agar within Q-Trays. Resultant colonies were picked and added to GM17 within Genetix 96-well plates (Genetix X6011), incubated overnight at 30°C before storage at –80°C in 44% glycerol (Sigma).

**Table 2:** Oligonucleotides used in this study.

Oligonucleotide	Sequence
NisAXXXHingeFor	5'- PHO TGATGGGTTGTNNKNNKNNKACAGCAACTTGTCATTGTAGT -3'
NisAXXXHingeRev	5'- CAAGTTGCTGTMNNMNNMNNACAACCCATCAGAGCTCCTGT -3'
pCI372Rev	5'- ACCTCTCGGTTATGAGTTAG -3'
For Primer (AAA)	5'- GTTGTGCTGCGGCAACAGCAACTTGTCATTGTAGTATTCAC -3'
Rev Primer (AAA)	5'- CAAGTTGCTGTTGCCGCAGCACAACCCATCAGAGCTCCTGT -3'
AAA Check Primer For	5'- CTGATGGCTTGTGCTGCGGCA -3'
SAA HC For	5'- TGATGGGTTGTTCAGCGGCTACAGCAACTTGTCATTGTAGT -3'
SAA HC Rev	5'- GCTGTAGCCGCTGAACAACCCATCAGAGCTCCTGTTTTACA -3'
SAA Forward SLT Codon	5'- TGATGGGTTGTTCGGCGGCTACAGCAACTTGTCATTGTAGT -3'
SAA Reverse SLT Codon	5'- GCTGTAGCCGCCGAACAACCCATCAGAGCTCCTGTTTTACA -3'
GGG Forward Primer	5’ GATGGGTTGTGGAGGTGGAACAGCAACTTGTCATTGTAGTA ‘3
GGG Reverse Primer	5’ AGTTGCTGTTCCACCTCCACAACCCATCAGAGCTCCTGTTT ‘3
GGG Check Primer	5’ TCTGATGGGTTGTGGAGGTGGA ‘3

PHO – 5’- Phosphate modification. Emboldened – Degenerate codons, emboldened & underlined – locations for site-directed mutagenesis.

### Site-directed mutagenesis of the nisin A ‘hinge’ region

The creation of targeted changes was also facilitated using a PCR-based approach, using oligonucleotides (Table 2) and a suitable pDF05 based template (one which most closely resembles the desired change). Amplification was through a 50 µl PCR reaction containing 0.02 U/ µl KOD Hot-Start Polymerase (Novagen), 1.5 mM MgSO_4_, 0.2 mM of each dNTP, 0.3 µM of both oligonucleotides and approximately 10 ng of template. Cycling conditions were as described above. Engineered plasmids were introduced into *L. lactis* NZ9800▵*nisA* via *E. coli* Top10 as described above.

### Deferred antagonism assays

Deferred antagonism agar-based assays were employed to assess the bioactivity of nisin derivative-producing strains [17]. Briefly, the *L. lactis* producers were ‘spotted’ (approximately 3 µl) onto GM17 agar and incubated for 16 hours at 30°C. In the case of *M. smegmatis,* the *L. lactis* producers were incubated for 40 hours. Growth media (0.75% agar) appropriate for growth of the individual target was seeded (0.5%) and poured over the *L. lactis* producers followed by further incubation at conditions suitable for the indicator. Enhancement in bioactivity was indicated by increased zone of inhibition relative to that generated by the wild type producer.

### Agar well diffusion assays

50 µl volumes of purified peptide were added to wells bored in the appropriate agar-containing media (1.5% agar, 0.75% for *M. smegmatis*) seeded with 0.5% of a 16 hour culture (40 hour culture for *M. smegmatis*) of the indicator of interest. Plates were incubated for 16 hours (40 hours for *M. smegmatis*) and bioactivity assessed on the basis of the size of the zone of inhibition.

### Identification of Nisin A derivatives

Colony mass spectrometric analysis was carried out on colonies exhibiting enhanced bioactivity using an Axima CFR plus MALDI TOF mass spectrometer (Shimadzu Biotech, Manchester, UK) and analyzed in positive-ion reflectron mode as previously described [8]. The changes to the *nisA* genes within the corresponding pDF05 derivatives were established through DNA sequencing (MWG, Biotech, Germany). The sequences for AAA, AAK, NAI, SAA and SLS were deposited in GenBank under the accession numbers KF664587, KF664588, KF664589, KF664590 and KF664591 respectively.

### Purification of nisin and derivatives thereof

2 litres of Tryptone Yeast (TY) broth were incubated for 20 hours with 20 ml of an overnight culture of producing strain. This culture was centrifuged for 20 minutes @ 8630g. The supernatant was decanted and passed through 60 g of pre equilibrated Amberlite XAD16 beads (Sigma-Aldrich). The beads were washed with 500 ml 30% ethanol and eluted with 500 ml 70% isopropanol (IPA) (Fisher) 0.1% trifluoroacetic acid (TFA) (Sigma-Aldrich). Concomitantly, the cell pellets were resuspended in 300 ml of 70% IPA 0.1%TFA and stirred at room temperature for 3 hours followed by centrifugation. This cell supernatant was combined with that referred to above and concentrated through rotary-evaporation (Buchi, Switzerland) to approximately 250 ml. After the pH was adjusted to 4.5 further concentration was achieved through the use of a Phenomenex SPE C-18 column to a final volume of 60 ml. 7 ml of this sample was concentrated, through rotary evaporation, to 2 ml and purified through HPLC using a Phenomenex C12 Reverse-Phase (RP) HPLC column (Jupiter 4 µ proteo 90 Å, 250 X 10.0 mm, 4 µm). To facilitate this, a gradient of 30–50% acetonitrile (Fisher) containing 0.1% TFA was developed. The relevant fractions were collected and pooled, subjected to rotary-evaporation to remove acetonitrile and freeze-dried (LABCONCO). The purified peptides were subjected to MALDI-ToF Mass Spectrometric analysis to confirm their purity before use.

### Minimum Inhibitory Concentration (MIC) Assays

Minimum inhibitory concentration assays were carried out in triplicate using 96-well plates (Sarstedt) pretreated with bovine serum albumin (BSA) as previously described [26]. Wild type nisin and nisin derivatives were adjusted to a 500 nM (when using *L. lactis* as a target) or 7.5 µM (all other indicators) starting concentration and 2-fold serial dilutions of each peptide were carried out. An overnight of the target strain was subcultured and incubated to an OD600 nm of 0.5 before being diluted to give a final inoculum of 10^5^cfu/ml in 200μl. The plates were incubated at an appropriate temperature and inspected after 16 hours. The MIC was determined as the lowest concentration at which no growth was visible.

## Results

### Creation of a bank of producers of randomised hinge derivatives

In order to fully exploit the potential of the nisin ‘hinge’ region (N20-M21-K22) to generate enhanced derivatives, it was decided to undertake a complete randomisation of this area. Using NNK scanning of the nisin A structural gene (*nisA*), a large bank of *L. lactis* NZ9800 pCI372*nisA* (pDF05) hinge variants were produced. In order to obtain full coverage with a 95% confidence limit using NNK scanning on three positions, a bank of 341,601 variants would have to be screened [27]. Creating and screening a bank of this size against four indicators would not have been feasible and thus a more practical bank of 12,000 variants was produced in order to increase the likelihood of finding interesting candidates in a short/medium time frame.

### Identification of nisin derivative producers with enhanced bioactivity

The bank of producers of randomised hinge derivatives was screened using deferred antagonism agar diffusion assays to identify producers that display enhanced bioactivity. The term bioactivity, as used here and elsewhere [8], [17], [19], reflects the overall activity of producer strains and does not discriminate between effects due to increased/decreased specific activity, altered peptide production levels, or effects on other physico-chemical properties such as diffusion in agar. Enhanced bioactivity was characterised by zones of clearing greater than that generated by the corresponding nisin A producing control against *Lactococcus lactis* HP, *Streptococcus agalactiae* ATCC 13813, *Mycobacterium smegmatis* MC2155 or *Staphylococcus aureus* RF122. From this screen 63 potentially enhanced producers were selected for further investigation and, after DNA sequencing, it was established that these corresponded to 23 unique mutants (Table 3).These 23 derivatives contained hinge regions consisting of combinations of 12 distinct amino acids (Table 4), none of which were aromatic or negatively charged in nature. Of these 12, alanine was most common (22%), followed by serine (16%) and glutamine (12%). To exclude that a bias or over-presentation of specific residues had occurred, and to insure the overall randomisation of the bank, 20 clones were chosen randomly and their hinge region was sequenced. The results are presented in Table 5 and establish that alanine and serine are not considerably over-represented within the bank. From the agar diffusion assays it was apparent that three producers consistently produced large zones of inhibition and these were selected for further investigation. DNA sequencing and mass spectrometric analysis revealed that the derivatives produced by these strains contained hinge regions consisting of AAK, SLS and NAI (Table 6). Assays with four target microorganisms established that the producer of the AAK-containing derivative exhibited enhanced bioactivity against *L. lactis* HP, *S. aureus* RF122 and *M. smegmatis* MC2155, the producer of the SLS-containing derivative exhibited enhanced bioactivity against *L. lactis* HP and *M. smegmatis* MC2155 and the producer of the NAI-containing derivative exhibited enhanced bioactivity against *L. lactis* HP, *S. agalactiae* ATCC 13813 and *M. smegmatis* MC2155. This enhancement could potentially be as a result of enhanced production, enhanced specific activity or some other enhancement with respect to the attributes of the peptide. To investigate these various possibilities, the three peptides produced by these strains (and wild type nisin A) were purified through HPLC and broth-based specific activity and agar diffusion assays were performed (Table 6). Against *L. lactis* HP, all three bioengineered peptides displayed enhanced activity, relative to equal concentrations of nisin A, when assessed through agar diffusion assays. However, this was not due to enhanced specific activity as broth-based MIC assays revealed that the activity of the NAI-containing peptide was equal, and those of the AAK- and SLS-containing peptides were reduced, relative to that of nisin A against this target (Table 6). All of the nisin derivatives displayed specific activity in broth which was reduced relative that of the wild type peptide against *S. aureus* RF122, yet the AAK- and NAI-containing peptides exhibited enhanced activity when assessed through agar diffusion assays, with the enhanced activity of the AAK-containing peptide being most significant (Table 6). When tested against *S. agalactiae* ATCC 13813, the NAI-containing peptide displayed significantly enhanced activity against this target in agar diffusion assays and in broth based specific activity assays. Regardless of assay, neither the AAK- nor the SLS-containing peptides exhibited enhanced activity against *S. agalactiae* ATCC 13813 (Table 6). *M. smegmatis* MC2155 was also included as an indicator as *M. smegmatis* is frequently used as a model microorganism/substitute for slow-growing, pathogenic mycobacteria. The AAK-containing peptide and the SLS-containing peptides both showed significant enhanced activity against this target in agar diffusion assays (Table 6), but the aggregative nature of MC2155 in broth precluded the generation of consistent MIC data from broth-based studies.

**Table 3:** Nisin A ‘hinge’ variants with enhanced bioactivity identified through the initial screen.

Variant	Molecular Mass	Variant	Molecular Mass
HVS	3304	AIT	3266
MAQ	3311	QVQ	3336
ASS	3226	SMT	3300
PVN	3291	HSQ	3332
ASV	3238	HAA	3260
HLA	3301	SIN	3294
NAI	3279	PQK	3334
ANP	3263	AQV	3278
SLS	3268	HSQ	3332
AAI	3236	PNA	3262
AAK	3250	NQV	3321
		HLS	3308

The three letter code corresponds to the amino acids located at each of the three ‘hinge’ sites.

**Table 4:** Frequency with which amino acids are located at each hinge site among the derivatives presented in Table 3.

Position 20 (% frequency)	Position 21 (% frequency)	Position 22 (% frequency)
Alanine 7 (30.4)	Alanine 5 (21.7)	Serine 4 (17.4)
Histidine 6 (26.1)	Serine 4 (17.4)	Glutamine 4 (17.4)
Proline 3 (13.0)	Valine 3 (13.0)	Alanine 3 (13.0)
Serine 3 (13.0)	Leucine 3 (13.0)	Valine 3 (13.0)
Asparagine 2 (8.7)	Glutamine 3 (13.0)	Isoleucine 2 (8.7)
Glutamine 1 (4.3)	Asparagine 2 (8.7)	Asparagine 2 (8.7)
Methionine 1 (4.3)	Isoleucine 2 (8.7)	Lysine 2 (8.7)
	Methionine 1 (4.3)	Threonine 2 (8.7)
		Proline 1 (4.3)

**Table 5:** Actual and expected frequencies of hinge amino acids from randomly selected representatives of the hinge mutant bank.

Amino Acid Residue	Actual Frequency (%)	Expected Frequency (%)
Serine	8.33	9.38
Alanine	6.67	6.25
Asparagine	6.67	3.13
Lysine	6.67	3.13
Tyrosine	6.67	3.13
Arginine	5.00	9.38
Glutamine	5.00	3.13
Glycine	5.00	6.25
Histodine	5.00	3.13
Isoleucine	5.00	3.13
Methionine	5.00	3.13
Stop	5.00	3.13
Valine	5.00	6.25
Aspartic Acid	3.33	3.13
Cytosine	3.33	3.13
Glutamic Acid	3.33	3.13
Leucine	3.33	9.38
Proline	3.33	6.25
Threonine	3.33	6.25
Trytophan	3.33	3.13
Phenylalanine	1.67	3.13

**Table 6:** Deferred Antagonism and Specific Activity Results.

		Deferred Antagonism			100 mg L^−1^ Agar Diffusion				MIC
		Zone Area (mm^2^)	p-value	Relative to WT (%)		Zone Area (mm^2^)	p-value	Relative to WT (%)	Specific Activity as % of WT
*L. lactis* HP	WT	299.58±7.04			267.69±9.21				
	AAA	492.83±40.27	0.012	164.51	416.00±17.99		0.001	155.41	100
	AAK	408.89±18.72	0.005	136.49	320.08±14.20		0.009	119.57	50
	SAA	483.14±33.89	0.009	161.28	344.76±18.50		0.008	138.71	50
	SLS	370.75±28.28	0.042	123.76	321.93±22.43		0.038	120.26	25
	NAI	439.52±47.88	0.034	146.71	371.32±19.60		0.004	128.79	100
*S. aureus* RF122	WT	138.31±3.36			99.34±12.33				
	AAA	292.24±11.46	0.001	211.29	155.77±3.30		0.011	156.81	25
	AAK	292.44±10.36	0.001	211.43	178.23±13.39		0.002	179.42	50
	SAA	236.28±13.53	0.004	170.83	130.60±4.46		0.036	131.47	25
	SLS	153.93±7.15	0.045	111.29	98.82±10.14		0.957	99.47	<6
	NAI	16.3.23±10.95	0.049	118.02	129.75±11.36		0.035	130.61	25
*S. agalactiae* ATCC 13813	WT	226.55±2.96			246.31±2.74				
	AAA	401.32±15.62	0.002	177.14	305.58±2.39		0.000	124.06	200
	AAK	232.00±21.58	0.706	102.41	234.55±4.23		0.021	95.23	50
	SAA	407.98±18.452	0.003	180.08	301.62±8.18		0.004	122.46	100
	SLS	209.45±17.11	0.223	92.45	226.05±4.10		0.003	91.78	25
	NAI	318.81±7.915	0.034	140.72	291.52±8.46		0.007	118.36	200
*M. smegmatis* MC2155	WT	51.31±10.08			67.79±4.308				
	AAA	277.65±60.42	0.02	541.09	149.24±4.776		0.000	220.15	ND
	AAK	199.47±32.36	0.009	388.73	105.73±6.930		0.003	155.96	ND
	SAA	303.85±59.93	0.016	592.15	101.19±11.510		0.026	149.27	ND
	SLS	230.31±20.31	0.001	448.84	119.56±8.59		0.003	176.36	ND
	NAI	270.04±58.30	0.02	526.26	78.50±8.16		0.137	115.8	ND

The zone of inhibition is expressed as the area of the zone of inhibition minus the area of the ‘spot’ in mm^2^. MIC: Minimum Inhibitory Concentration. ND: Not Determined.

### Rational design of nisin derivatives with enhanced bioactivity

The amino acid composition of nisin hinges within strains exhibiting enhanced bioactivity revealed some trends. All ‘improved’ hinge regions had a mass less than that of the wild type, alanines were frequently identified at each position and, on a number of occasions, multiple alanines were present. Based on these observations, it was postulated that a nisin derivative with a hinge consisting of AAA could potentially display enhanced properties. The presence of a serine, particularly at position 20, in the nisin hinge of other strains displaying enhanced bioactivity has also been noted, including hinges consisting of SMT and SLS from this study and SVA from Rouse et al. [17]. On the basis of this observation, a nisin derivative containing a SAA hinge was also created. To further test the theory that nisin derivatives with a hinge consisting of small amino acids may exhibit enhanced features, site-directed mutagenesis was also used to create a hinge derivative consisting of glycine residues only. In this last case, the producer of the GGG-containing hinge did not exhibit antimicrobial activity. It would thus seem that a lack of chirality within the hinge is a negative feature.

In contrast, the respective producers of the AAA- and SAA-containing peptides displayed enhanced bioactivity against each of the four strains tested and again were purified for specific activity assays (Table 6). Both the AAA- and SAA-containing peptides showed significantly enhanced specific activity relative to nisin A against all four indicators in agar diffusion assays. In each case, this enhancement was more significant in the case of the AAA-containing peptide. In contrast, in broth based specific activity assays the bioengineered peptides exhibited activity that was equal or reduced relative to that of the wild type peptide, with the exception that the AAA-containing peptide showed a two-fold improvement relative to the natural peptide against *S. agalactiae* ATCC 13813. Thus, while in many cases it would appear that enhanced bioactivity is attributable to enhanced diffusion through complex media, a phenomenon previously reported by Rouse et al. [17], this result establishes that antimicrobial potency can also be a contributory factor with respect to some targets.

## Discussion

In this study a large bank of derivatives of the lantibiotic nisin was generated in which all three hinge residues were simultaneously randomised. To facilitate the relatively rapid screening of such a large number of derivative-producing strains, the deferred antagonism assay was employed. This approach allows for the identification of variants with enhanced bioactivity and can be supported by further assays to determine if this enhanced bioactivity is attributable to enhanced specific activity [15], [25] production and/or solubility/diffusion [17]. Regardless of the specific underlying basis for the enhanced bioactivity of a particular derivative, any enhanced feature has the potential to be exploited in food or medicine. This is especially true when one considers the number of applications which are available to lantibiotics, and nisin in particular [1].

Preliminary screening of this bank resulted in the identification of 23 derivative producers with enhanced bioactivity. Of these 23, producers of nisin derivatives containing SLS, AAK or NAI within the hinge region were brought forward for further characterisation. In the case of the SLS-containing example, the selection of a strain producing nisin with a serine (S) at positions 20 and 22 was consistent with observations made by Field et al. [8] and Rouse et al. [17], who previously established that the introduction of this hydrophilic amino acid at position 22 and 20 can result in enhanced bioactivity. Field and co-workers have also previously noted that the introduction of a leucine (L) at position 21, in several instances, lead to relatively high levels of activity. With respect to the NAI-containing derivative, nisin A naturally contains an asparagine (N) at position 20, the benefits of incorporating alanines will be discussed in greater depth below and, although there is no precedent for an enhanced derivative containing an isoleucine (I) at position 22, its introduction at the other two hinge positions has had varying effects on bioactivity [8]. The introduction of alanines in the AAK-containing peptide will be discussed below.

Although this is not the first study which has fully randomised the nisin ‘hinge’ region, it is the first to do so in the context of the full length nisin peptide. Previously, Plat and co-workers [28] randomised all three positions in a truncated form of nisin, i.e. nisin-(1–22), and found when they analysed 16 of the active derivatives that the size of the zone was directly, for the most part, proportional to the amount of the prepeptide produced. However, in that instance the derivatives that exhibited the largest zones of activity both contained the aromatic amino acid tryptophan (W); AWR and WRA. In contrast, in this study none of the peptides produced by the ‘hinge’ mutants that displayed enhanced bioactivity contained aromatic amino acids. The differences between the composition of the hinges in enhanced peptides from these respective studies suggest that the hinge is performing a different role in the truncated, relative to the intact, peptide and that in the latter instance the impact on the hinge residues on the C terminal domain is critical. Despite these differences, it is apparent that there is a consistent absence of negatively charged amino acids from within the hinge of all peptides and strains exhibiting enhanced bioactivity. This is also consistent with the previous studies of Field et al. [8] and Yuan et al. [18]

Well diffusion assays using purified forms of the SLS-, AAK- and NAI-containing peptides established that enhanced bioactivity was attributable to an enhanced diffusion in agar in a manner similar to that previously reported by Rouse et al. [17]. This trait was previously noted as being a valuable one in that such a peptide performed better than wild type nisin A with respect to controlling *Listeria monocytogenes* in a food system. The identity of these, and other, changes that occurred in the hinge region of peptides associated with enhanced bioactivity in the preliminary screening also provided a further insight into the flexibility of the hinge and revealed distinct patterns (Tables 3 and 4). The frequency with which alanine appears was particularly notable. Among the strains that exhibited enhanced bioactivity, alanine was the amino acid that was most frequently located at positions 20 (30%) and 21 (22%) and was also frequently identified at position 22 (13%). It was also noted that a pair of alanines was located in the hinge region of peptides from three strains exhibiting enhanced bioactivity. The AAK-containing peptide is a perfect example of this pattern. The third residue, lysine, is conserved across all natural variants of nisin; nisin A [29], nisin Z [30], nisin Q [31], nisin F [32], nisin U and U2 [33] and most recently nisin P [34]. The enhanced bioactivity of the corresponding strain and the specific activity in agar of the corresponding peptide was particularly apparent against *S. aureus* RF122 (Table 6). Despite the conserved nature of this lysine, this study and others [8], [17] have demonstrated that this residue can be changed. On the basis of these observations it was decided to bioengineer a strain to produce a peptide in which the ‘hinge’ would consist wholly of alanines. The fact that these changes enhanced the bioactivity of the associated strain means that this is the first instance upon which a characteristic of nisin has been enhanced through rational design. The additional hinge derivative to result from rational design contained a hinge consisting of SAA. The creation of this peptide was targeted due to the previous observation that enhanced bioactivity was evident in strains that produced peptides with a serine at position 20 of the nisin peptide, including SMT, SLS and SVA [17]. The bioactivity of the resultant strain (Table 6) was also greater than that of nisin wild type producer against all indicators tested. The fact that the newly introduced serine remained unmodified is in agreement with observations made previously by Lubelski [35], where it was suggested that serines in positions immediately preceding lanthionine bridges remain unmodified. The fact that the addition of small chiral amino acids to the hinge resulted in an increase in bioactivity may be attributable to an increase in ‘hinge’ flexibility. In contrast, the production of the achiral glycine ‘hinge’ may confer a structurally weak hyper flexible ‘hinge’ lacking in any distinguishable conformity through mis-folding. It should also be noted that, while we focused on the observation that alanine and serine were frequently identified across all hinge residues in strains with enhanced bioactivity, there may be merit in designing residues whereby the amino acid at each respective location is optimised. Indeed, the positively charged histidine was found very frequently at position 19 in these strains while, at position 20, the hydrophobic residues valine, leucine and isoleucine are very prominent. The identity of the residues located at position 22 was more variable.

Ultimately, the ‘hinge’ region of nisin has again been established to be a worthy target with respect to bioengineering to enhance bioactivity. The benefits of incorporating small chiral amino acids were particularly apparent leading to, for the first time, the rational design of nisin ‘hinge’ derivatives with enhanced properties.
